# Advances in Pharmacokinetic Mechanisms of Transporter-Mediated Herb-Drug Interactions

**DOI:** 10.3390/ph15091126

**Published:** 2022-09-08

**Authors:** Jie Li, Shuting Wang, Fengjie Tian, Shuang-Qing Zhang, Hongtao Jin

**Affiliations:** 1 New Drug Safety Evaluation Center, Institute of Materia Medica, Chinese Academy of Medical Sciences & Peking Union Medical College, Beijing 100050, China; 2 Beijing Union-Genius Pharmaceutical Technology Development Co., Ltd., Beijing 100176, China; 3 NMPA Key Laboratory for Safety Research and Evaluation of Innovative Drug, Beijing 102206, China; 4 Chinese Center for Disease Control and Prevention, National Institute for Nutrition and Health, 29 Nanwei Road, Beijing 100050, China

**Keywords:** drug transporters, herbs, herb–drug interactions, pharmacokinetics

## Abstract

As the use of herbs has become more popular worldwide, there are increasing reports of herb-drug interactions (HDIs) following the combination of herbs and drugs. The active components of herbs are complex and have a variety of pharmacological activities, which inevitably affect changes in the pharmacokinetics of chemical drugs in vivo. The absorption, distribution, metabolism, and excretion of drugs in vivo are closely related to the expression of drug transporters. When the active components of herbs inhibit or induce the expression of transporters, this can cause changes in substrate pharmacokinetics, resulting in changes in the efficacy and toxicity of drugs. In this article, the tissue distribution and physiological functions of drug transporters are summarized through literature retrieval, and the effects of herbs on drug transporters and the possible mechanism of HDIs are analyzed and discussed in order to provide ideas and a reference for further guiding of safe clinical drug use.

## 1. Introduction

Herbs have been used to prevent and treat diseases for thousands of years. Since the 1990s, the use of herbal products has risen, and this trend may continue. People are eager to return to nature for medical care and believe that herbs extracted from natural raw materials are safe. Particularly in rural areas, the availability and affordability of herbs have greatly improved their utilization [1]. According to WHO estimates, by 2050, the global market for herbal products will reach approximately $5 trillion [2]. The widespread use of herbs has greatly increased the likelihood of their concomitant use with chemical drugs. Furthermore, many patients with complex conditions also need to be treated with a combination of herbs and chemical drugs. Combination therapy is commonly used to achieve a better therapeutic effect than single therapy. For example, it has been found that the combination of turmeric and metformin can produce beneficial pharmacokinetic and pharmacodynamic interactions, thus improving the hypoglycemic and hypolipidemic effects. *Ginger* can potentiate the antiplatelet aggregation effect of nifedipine in normal volunteer and hypertensive patients [3]. However, in addition to the benefits of enhancing efficacy and reducing toxicity there are also many studies that have found undesirable side effects when herbs are used in combination with certain chemical drugs. For example, the combination of St. John’s wort and cyclosporine (CSP) reduces the plasma concentration of CSP in transplant patients and leads to acute rejection [4,5,6,7]. Some herbs (*Ginkgo biloba, Salvia miltiorrhiza*) can enhance the activity of the platelet inhibitor aspirin, leading to clinical bleeding events [8,9]. Rational use of herb-drug interactions (HDIs) will improve drug efficacy and reduce toxicity, but HDIs may also produce unexpected adverse reactions. Therefore, understanding the mechanism of HDIs to improve the safety and effectiveness of combination therapy has become very important.

The occurrence of HDIs is usually related to the changes in pharmacodynamics and pharmacokinetics caused by herbs. Chemical pharmacodynamics and pharmacokinetics are closely related to the expression of transporters. Drug transporters are proteins widely expressed in various tissues and organs, which mediate the transmembrane transport of substrates and play a key role in the absorption, distribution, metabolism, and excretion of drugs. Herbs may affect drug concentrations in plasma and tissues by inducing or inhibiting the expression of transporters. This article focuses on tissue distribution and physiological function of drug transporters and discusses and analyzes transporter-mediated HDIs in order to clarify the mechanism of transporter-mediated HDIs, predict the possible benefits and risks of mutual drug use, and provide a reference for safe and effective clinical drug use.

## 2. Tissue Distribution and Physiological Function of Transporters

At present, approximately 400 types of membrane proteins have been identified in the human body, which are divided into two superfamilies: the solute carrier (SLC) superfamily and ATP binding cassette (ABC) superfamily [10].

The SLC superfamily includes 52 different gene families involving more than 300 membrane-binding proteins [11]. The SLC superfamily includes organic cation transporters (OCTs/SLC22As), carnitine transporters (OCTNs/SLC22As) [12], mammal multidrug and toxin extrusion proteins (MATEs/SLC47As), organic anion transporters (OATs/SLC22A), peptide transporters (PEPTs/SLC15As) [13], and the organic anion transporting polypeptides (OATPs/SLCOs) [14]. These SLC transporters, which are widely distributed in various tissues, such as the intestine, liver, kidney, and brain, are mainly involved in the uptake of small molecules (MATE transporters are responsible for the efflux of substrates). They mediate the movement of solute from the extracellular milieu into cells, either by passive diffusion along its concentration gradient, by cotransport, or counter-transport against its concentration gradient by co-opting the concentration gradient of another solute. The gradient is ultimately derived from ATP hydrolysis-driven transporters using ATP as energy. Thus, SLC transporters are considered facilitated transporters or secondary active transporters. They regulate the transport of various essential molecules (such as inorganic ions, sugars, amino acids, neurotransmitters, and drugs) through a variety of mechanisms. [15].

OCTs found in human species include OCT1 (SLC22A1), OCT2 (SLC22A2), and OCT3 (SLC22A3). The membrane topology of OCT isoforms is predicted to be similar with 12 α-helical transmembrane domains with intracellular amino and carboxy termini. An extracellular loop between transmembrane domains 1 and 2 contains a potential N-glycosylation site. A large intracellular loop resides between transmembrane domains 6 and 7 and possesses predicted phosphorylation sites. OCTs are uptake transporters that control the cellular entry of small, positively charged compounds, including drug substrates, such as the platinum-containing antineoplastics, the antidiabetic metformin, and the histamine H2 receptor [14,16].

OCTNs transport carnitine, whereas OCTN1 (SLC22A4) protein is predicted to contain 11 transmembrane domains and one nucleotide-binding domain, OCTN2(SLC22A5) probably has 12 transmembrane domains. OCTN1 is an organic cation uniporter or H/organic cation antiporter that can transport in both directions. OCTN2 can act as organic cation uniporters or sodium-carnitine cotransporters. Both OCTN1 and OCTN2 transport tetraethylammonium, verapamil, quinidine, ergothioneine, and pyrilamine. OCTN2 also transports the antiseizure drug valproic acid, the antibiotic cephaloridine, and the diuretic spironolactone [16].

MATEs found in the human species include MATE1 (SLC47A1) and MATE2-K (SLC47A2). MATE1 and MATE2-K are composed of 13 putative transmembrane domains with amino and carboxyl termini on the intracellular and extracellular faces of the plasma membrane, respectively. MATE1 effluxes organic cations such as tetraethylammonium, 1-methyl-4-phenylpyridinium, oxaliplatin, and paraquat, using a proton-coupled electroneutral exchange. MATE2-K transports organic cations, including tetraethylammonium, 1-methyl-4-phenylpyridinium, cimetidine, procainamide, and metformin [16].

OATs move small organic anions against their concentration gradient using a Na^+^ gradient maintained by Na^+^/K^+^-ATPase. OAT transporters have 12 predicted transmembrane domains arranged in two sets of six helical domains. OATs are thought to have two large loop structures between transmembrane domains 1 and 2 and domains 6 and 7. The first loop is extracellular and contains glycosylation site. Glycosylation at multiple sites often results in a range of molecular weights reported for OAT transporters. The second loop occurs intracellularly and contains phosphorylation sites of particular importance in drug disposition are OAT1 (SLC22A6) and OAT3 (SLC22A8). OAT substrates include steroid hormones, biogenic amines, and drugs such as the angiotensin-converting enzyme inhibitors captopril and quinaprilat, the angiotensin II receptor blocker olmesartan, and numerous antibiotics and antivirals [14,16].

PEPTs are proton-coupled transporters, and one isoform, PEPT1, is often studied. PEPT transporters are predicted to have 12-α-helical transmembrane domains with a large extracellular loop between domains 9 and 10 and intracellular carboxyl and amino termini. PEPT1 (SLC15A1) can specifically transport dipeptide and tripeptide amino acids. Drugs or prodrugs with sufficient spatial similarity to dipeptides and tripeptides can also be used as potential substrates of PEPT1, such as β-lactam antibiotics, protease inhibitors and antiviral drugs (valacyclovir) have high affinity with PEPT1 [16,17].

OATPs found in human species include OATP1B1 (SLCO1B1), OATP1B3 (SLCO1B3), OATP2B1 (SLCO2B1). OATPs are integral membrane proteins predicted to contain 12 transmembrane helices with amino and carboxyl termini oriented to the cytoplasmic face. A large extracellular domain is thought to be located between transmembrane domains 9 and 10, with N-glycosylation sites present in extracellular loops 2 and 5. OATPs have a wide substrate specificity for amphipathic molecules, including endogenous compounds such as conjugated metabolites of steroid hormones, bile acids, thyroid hormones, sulfated and glucuronidated hormones, and drug substrates including rifampicin, methotrexate, antidiabetics, and statins [14,16,18].

ABC transporters are composed of transmembrane domains containing typically 5 to 6 transmembrane helices and cytoplasmic ABC units or nucleotide-binding domains, which are responsible for the binding and hydrolysis of ATP. ABC proteins are present in all sorts of organisms; both uptake and efflux transporters (importers and exporters) can be found in prokaryotes, whereas ABC transporters in eukaryote plasma membrane are exclusively exporters [19]. The ABC superfamily uses the synthesis and hydrolysis of ATP to drive the transmembrane transport of various substrates such as ions and molecules, with the role of substrate efflux and efflux transporters, maintaining the level of endogenous (hormones, ions, and neurotransmitters) and exogenous (drugs and toxins) substances in cells, promoting the excretion of metabolites and toxins, and protecting the organism with complex network physiological functions from the absorption of harmful substances [20]. Members of the ABC superfamily are important in drug efficacy and toxicity, including P-glycoprotein (P-gp/ABCB1), breast cancer resistance protein (BCRP/ABCG2), multidrug resistance-associated proteins (MRPs/ABCCs), and the bile salt export pump (BSEP/ABCB11) [12,14,21]. 

P-gp is encoded by the human gene ATP binding box subfamily B member 1 (ABCB1) or multidrug resistance 1 (MDR1). It is an ATP-dependent efflux pump. The structural topology of P-gp consists of two distinct regions containing six putative transmembrane domains and one nucleotide-binding domain. The amino and carboxyl termini of P-gp are located intracellularly. P-gp has broad substrate specificity for structurally divergent compounds; in general, its substrates are hydrophobic and may be cationic. Substrates of P-gp include HIV protease inhibitors, calcium channel blockers, and anticancer drugs of the vinca alkaloid, anthracycline, and taxane classes. P-gp is inhibited by numerous compounds including verapamil, ritonavir, and CSP [14,16].

BCRP is an efflux transporter found in breast cancer cell lines. It is encoded by the human ABCG2 gene. The BCRP protein is considered a “half-transporter” consisting of two domains: an amino-terminal ATP-binding domain and carboxyl-terminal transmembrane domain (six transmembrane segments). BCRP substrates include numerous anticancer agents, such as the topoisomerase II inhibitor etoposide, the camptothecin derivatives topotecan and irinotecan, and the tyrosine kinase inhibitors imatinib and gefitinib. Other substrates of BCRP include statins, antibiotics, numerous environmental toxins, and endogenous substrates such as conjugated steroid hormones, folates, and uric acid [14,16].

Of the MRPs, MRP1 (ABCC1), MRP2 (ABCC2), and MRP4 (ABCC4) have been most widely studied in the context of drug response and toxicity. MRP transporters contain consensus regions named the Walker A, Walker B, and Signature C motifs that are required for ATP binding. MRP1 and -2 contain three membrane-spanning domains with a total of 17 hydrophobic transmembrane regions. For these two MRP proteins, computational analysis suggests extracellular and intracellular amino and carboxyl termini, respectively. MRP4 is a smaller protein with only two domains that span the plasma membrane (12 total transmembrane regions). The amino and carboxyl termini are both predicted to be intracellular for MRP4. MRPs have two intracellular nucleotide-binding domains. Endogenous substrates of MRP1 include oxidized glutathione, cysteinyl leukotrienes, glucuronide and sulfate conjugates, and drug substrates including anthracyclines, vinca alkaloids, and antivirals. MRP2 transports a wide range of glutathione, sulfate, and glucuronide-conjugated endo- and xenobiotics. Substrates of MRP4 include numerous endogenous compounds involved in cellular signalings, such as cyclic nucleotides, eicosanoids, urate, and conjugated steroids, as well as folate, bile acids, and glutathione. Drug substrates of MRP4 include cephalosporin antibiotics, nucleotide analog reverse transcriptase inhibitors, and cytotoxic agents such as methotrexate and 6-mercaptopurine [14,16].

BSEP is a 12-membrane-spanning domain protein containing putative glycosylation sites, nucleotide binding domains, and typical structures. As the primary canalicular bile acid transporter, BSEP primarily transports conjugated bile acids (including taurochenodeoxycholate, taurocholate, tauroursodeoxycholate, glycochenodeoxycholate, and glycocholate) in an ATP-dependent manner [16].

Some studies have found that P-gp, MRPs, and BCRP are highly associated with multidrug resistance (MDR) [22]. Their increased expression in cancer cells enhances the efflux of certain anticancer drugs. This results in the decrease or abrogation of their cytotoxic efficacy, thereby contributing to chemoresistance in various tumor cells, subsequently leading to increased tumor burden and reduced treatment outcomes [23].

**Figure 1:**
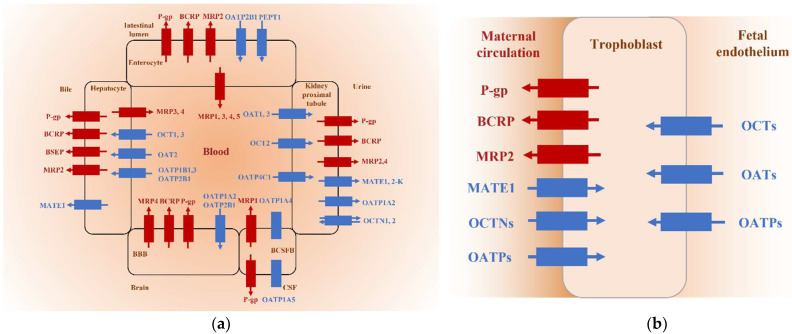
Expression of ABC (red) and SLC (blue) transporters with major roles in drug efficacy or toxicity in (**a**) human intestinal epithelia, hepatocytes, kidney proximal tubule epithelia, brain capillary endothelial cells, and choroid plexus epithelial cells; (**b**) trophoblast cell. Little is known about the expression and function of some drug transporters in the BCSFB system, therefore, directional arrows are not included.

Different subtypes of SLC and ABC drug transporters are widely distributed in epithelial cells (apical or basolateral membrane) of various tissues in the body and are especially highly expressed in some important organs of the human body, such as organs with absorption and excretion functions (liver, kidney, and intestine) and organs with barrier functions (brain, placenta, and retina) (Figure 1) [24]. SLC transporters and ABC transporters often share tissue distributions and overlap substrate specificities. The interplay of the uptake transporters and efflux transporters together with phase I or II metabolism may be required for the sequential traverse of the basolateral and apical membranes. Therefore, drug transporters can be regarded as completing the phase I or II enzyme-based detoxification system; drug uptake delivers the drug to the detoxification system to facilitate metabolism, whereas drug efflux decreases the load on detoxification enzymes. For example, some efflux transporters (such as P-gp and MATEs) are mainly expressed on the luminal membranes of renal tubules, and uptake transporters (such as OCT2 and OATs) are often expressed at the basolateral membrane of renal tubules; they work in series to structure an eliminatory pathway of a particular drug via urine [14]. Here, we summarize the main tissue distribution and functions of transporters (Table 1). 

### 2.1. Intestinal Transporters

Absorption is the process of drugs entering the blood circulation from the administration site. Orally administered drugs must pass through the gut wall mucosa before reaching the capillaries that lead to the portal vein and enter the internal circulation [25]. Compared with intravenous administration, the bioavailability (extent and rate of absorption) of oral drugs has always been the focus of researchers. The absorption process of oral drugs may be affected by many physical and chemical factors and the body environment, such as the release rate of drug preparations, gastrointestinal motility, pH, biotransformation and intestinal wall permeability, which may affect the bioavailability of drugs. As one of the determinants of pharmacokinetics, intestinal transporters can affect the oral absorption of many drugs by ingesting drugs or pumping them into the intestinal lumen. It has been determined that several major transmembrane transporters such as P-gp, BCRP, MRP, PEPT, OCTs, and OATPs mediate drug transport in the intestines [26].

P-gp is one of the most widely studied transporters in the intestine which is expressed in the apical membrane of enterocytes. P-gp limits the passage of oral drugs (mainly lipophilic compounds) through the intestinal tract into the systemic circulation and plays an important role in regulating drug absorption [27,28].

BCRP and MRP2 have high protein expression levels on the apical (lumen) membrane of intestinal epithelial cells. They are distributed in the intestine and play a combined efflux role, pumping potential specific substrates harmful to the body back into the intestinal cavity. This also reduces the intestinal absorption of many clinically used drugs, such as anticancer drugs, immunosuppressants, statins and antibiotics, and limits the oral bioavailability of these drugs [25,29]. MRP1, MRP3, MRP4, and MRP5 are located in the basolateral membrane of intestinal epithelial cells. They participate in the process of transporting some drugs from intestinal epithelial cells to the blood, and promote the drugs to enter the blood circulation. For example, MRP3 and MRP4 participate in the basal lateral transport of cephalexin [30,31]. Therefore, for some drugs that require attention, it is necessary to evaluate the pharmacokinetic changes and potential HDIs caused by the effect of exogenous substances on the activity of their specific transporters.

PEPT1 is the most widely studied intestinal uptake transporter. It is widely expressed on the apical membrane of the duodenum, jejunum, and ileum. PEPT1 plays an important role in maintaining intestinal homeostasis and health. [17,32]

OATPs and OCTs are evenly distributed in the intestine. OCT1 and OATP2A1 are two with extensive function in the intestinal tract, however, their exact distribution requires further study. In recent years, several studies on the tissue distribution of OATP2B1 have resulted in different views on the distribution of OATP2B1 in the apical membrane and basement membrane of intestinal epithelial cells [26,33]. Some authors have identified the protein in the apical membrane of human enterocytes or Caco-2 cells by immunohistochemical analysis, which has been frequently used to explain the intestinal absorption of drugs such as fexofenadine, pravastatin, and talinolol [18,34]. But another study reported the basolateral localization of OATP2B1 and verified this finding by co-localization with P-gp, MRP2, and PEPT1, which were clearly identified in the opposite membrane [35]. However, according to the experimental results, the conclusion that OATP2B1 is located in the apical membrane is more reliable. In the experiment of co-localization with other transporters, OATP2B1 is located at the basolateral membrane, but where the IHC-staining of OATP2B1 is rather weak. There is also some controversy regarding the location of OCT1 in intestinal epithelial cells; however, it is generally believed that OCT1 is mainly located in the apical membrane of the intestine and mediates drug uptake on the luminal side of intestinal cells [24,25,26,27]. Many cationic drugs are in vitro substrates of OCT1, such as morphine, metformin, and trospium. The tissue distribution of drug transporters in the intestine requires further investigation.

### 2.2. Liver Transporters

The liver has the function of producing and secreting bile. It is an important organ in detoxification. Major liver uptake transporters, including OATP1B1, OATP1B3, OATP2B1, OAT2, OCT1, OCT3 and sodium taurocholate co-transporting polypeptide (NTCP), candrive liver uptake of a wide variety of drugs and metabolites. These transporters mediate the uptake of various endogenous compounds (such as bile salts) and exogenous compounds from the blood into hepatocytes, thus playing an important role in the distribution of drugs and endogenous compounds [36]. In the liver, OATP1B1, OATP1B3, OATP2B1, OCT1, and OCT3 are specifically expressed on the basolateral membrane of hepatocytes which mediate the absorption of exogenous and endogenous substances (bile acids and cholesterol) in the liver [37,38]. ABC transporters including P-gp, BCRP, MRPs and BSEP excrete cholesterol, bile salts and other metabolites in the liver [39]. P-gp is located in the tubular membrane of hepatocytes and the apical membrane of bile duct cells. It has a wide range of substrate specificity, primarily transporting hydrophobic neutral and cationic compounds, and excreting drugs into bile for clearance [39]. MRP3 and MRP4 are expressed in the basolateral membrane of hepatocytes, which have a high affinity for endogenous substances, such as bile acids and steroid conjugates, indicating that they have a protective role in preventing cholestasis and hepatotoxicity [37]. BCRP is distributed on the apical membrane of human hepatocytes and mediates the clearance of drugs and metabolites in the liver. [38]. MRP2 and BSEP are distributed on the apical membrane of human hepatocytes and mediate the clearance of drugs and metabolites in the liver. Some studies have shown that down-regulation of the functions of BSEP and MRP2 leads to the accumulation of toxic substrates in hepatocytes, which is one of the mechanisms of isoniazid-induced liver injury [40]. BSEP is the only transporter that mediates the secretion of bile salts into bile. It exports a restricted number of molecules, predominantly monovalent taurine- and glycine-conjugated bile salts. Impaired bile salt secretion caused by a lack or insufficient amount of functioning BSEP in the canalicular membrane may lead to intrahepatic cholestasis characterized by diminished bile production [19,36].

### 2.3. Renal Transporters

The kidney is the main excretory organ. Most free drugs or metabolites can be excreted from the urine through passive renal tubular filtration and active renal tubular secretion. The secretion and reabsorption of proximal tubules mediated by OATs and OCTs are the main way the kidney realizes material transport [41]. Transporters OAT1, OAT3, and OATP4C1 located in the basolateral membrane of renal tubules and MRP2, MRP4, OATP1A2, and BCRP on the renal tubular apical membrane mediate the uptake and efflux of organic anionic drugs. The transporter OCT2, located in the basolateral membrane of renal tubules, and MATE1, MATE2/2-k, P-gp, OCTN1, and OCTN2 on the apical membrane mediate the secretion of organic cationic drugs [42,43]. These transporters have multiple substrate specificity, and there is substrate overlap between transporters. When two or more drugs are involved in the transport system of renal tubules, the competitive inhibition of drug transporters may lead to the abnormal accumulation of drugs in renal tubular epithelial cells, resulting in unexpected drug-drug interactions (DDIs). Furthermore, an imbalance between transporter-mediated uptake and efflux may result in drug accumulation in proximal tubule cells, leading to drug-induced nephrotoxicity and kidney injury [44]. Therefore, people should pay more attention to DDIs and nephrotoxicity influenced by renal transporters [45].

### 2.4. Barrier Transporters

The blood-brain barrier (BBB) and blood-cerebrospinal fluid barrier (BCSFB) are protective tissue barriers that isolate the central nervous system from the systemic circulation. The BBB is composed of tight junctions of brain endothelial cells (BECs), while the BCSFB is composed of choroid plexus (CP) epithelial cells, which restricts the entry of toxins and other harmful metabolites into brain tissue.

In the BBB system, ABC efflux transporters P-gp, BCRP, and MRPs are abundantly expressed. They have a wide range of substrates, including many therapeutic drugs, such as antibiotics and anticancer agents, and restrict these drugs from entering the brain. In brain capillaries, P-gp is predominantly and abundantly expressed in the luminal membrane, pumping the substrate diffused in endothelial cells back to the blood [46]. Some β-lactam antibiotics, such as benzylpenicillin, ceftriaxone, and ampicillin, are substrates of P-gp, which might account for their low brain penetration. BCRP is expressed at the luminal membrane of human BECs which are involved in benzylpenicillin efflux transport in humans. MRP1 and MRP4 are expressed in human brain capillary endothelial cells, astrocytes, microglia, and the CP. They have been demonstrated to be associated with brain tumor resistance and they mediate the efflux transport of a wide range of drugs, HIV protease inhibitors, organic anions, prostaglandins, antibiotics, and nucleoside analogs. In addition, MRP2, MRP3, and MRP5 are also expressed in human brain capillary endothelial cells. ABC efflux transporters play a very important role at the BBB in protecting the brain; however, they are also an obstacle for therapeutic agents entering the brain. Therefore, breakthroughs in how to regulate the ABC transporters of the BBB to increase brain protection (up-regulate ABC transporters) or improve drug delivery to the brain (inhibit ABC transporters) are necessary. Some SLC transporters are also expressed in the BBB system. OATP1A2 and OATP2B1 are localized at the luminal membrane of BECs in humans to mediate drug absorption [46,47].

In the BCSFB system, P-gp located in the lumen membrane transports substrates back into the bloodcerebrospinal fluid (CSF). MRP1, the most abundant efflux transporter in the CP, is located in the basolateral membrane and mediates substrate transport with OAT1 and OAT3 [47], while OAT1 and OAT3 are found in epithelial cells of the human CP, but their precise localization is unclear. Cellular localization of two OATPs in the CP has been confirmed by immunochemical studies: OATP1A4 is located at the basolateral membrane, while OATP1A5 is located on the apical membrane, with OATP2 probably the most abundant OATP in the CP [46].

Human placenta is the feto-maternal interface during pregnancy and functions as the main barrier between maternal and fetal circulation. Placental trophoblast cells have the dual role of absorption and excretion in embryonic development. Drug transporters are expressed on the apical membrane and basolateral membrane of trophoblast cells, and function in the placental endocrine system and protect against foreign substances, acting as an important part of the placental barrier [12]. At present, the ABC efflux transporters widely studied in the placenta are P-gp, BCRP, and MRP2, which are distributed in the top membrane of trophoblast cells (facing the mother). The energy released by ATP hydrolysis is used to pump the substrate from the placenta back to the mother for circulation, in order to reduce exposure of the fetus to drugs and foreign substances [48]. Studies have shown that etravirine inhibits BCRP-mediated substrate efflux and increases the concentration of nucleotide reverse transcriptase inhibitors (tenofovir and abacavir) in fetuses [49]. Therefore, the introduction of etravirine into antiretroviral therapy containing tenofovir should be avoided during pregnancy to protect the safety of the fetus. SLC transporters expressed in the placenta, including OCTs, OATs, OCTNs, OATPs, and MATE1 are located on both sides of trophoblast cells and facilitate energy-independent uptake of hydrophilic or charged molecules by trophoblast cells which play an important role in maintaining the stability of the internal environment of the placenta. The expression and activity of drug transporters in the placenta will change during pregnancy and are closely regulated by many factors. In addition, due to the overlapping specificity of placental drug transporters in the substrate spectrum, the increased or decreased expression of one placental drug transporter may not necessarily affect the role of drugs in the placental barrier, and other transporters may compensate. More preclinical and clinical studies are needed on the expression of placental transporter function and the use of drugs during pregnancy [12].

**Table 1:** Classification and the main distribution and functions of common transporter.

Classification		Common Transporters	Main Distribution in Human	Function	References
SLC superfamily	OCTs	OCT1	liver, brain	Mediate the uptake of hydrophilic or charged molecules, including endogenous (sugars, amino acids, nucleosides, neurotransmitters) and exogenous (drugs)	[12,24,25,42]
		OCT2	kidney, brain		
		OCT3	widely distributed in various organs		
	OATs	OAT1	liver, kidney, brain, placenta		[46]
		OAT2	liver, kidney, brain		
		OAT3	liver, kidney, brain, skeletal muscle		
		OAT4	liver, kidney, brain, placenta		[12]
	OATPs	OATP1A2	liver, kidney, brain		[12,26,33,38,46]
		OATP2A1 OATP3A1 OATP4A1	widely distributed in various organs		
		OATP1C1	brain		
		OATP4A1	placenta		
		OATP2B1	liver, placenta, ciliary body		
		OATP1B1 OATP1B3	liver		
		OATP4C1	kidney		
	PEPTS	PEPT1	intestines, brain, placenta		[17]
	OCTNs	OCTN1	liver, placenta	Mediate the secretion of organic cationic drugs	[12]
		OCTN2	liver, brain, placenta		
	MATE Transporters	MATE2/2-K	kidney	Mediate the elimination of hydrophobic exogenous molecules (drugs, toxins, metabolites, conjugates)	[42]
		MATE1	liver, kidney, placenta		
ABC superfamily	P-gp	P-gp	intestines, liver, kidney, placenta		[27,28,39,46,48]
	BCRP	BCRP	intestines, liver, kidney, brain, placenta		[39,46,49]
	MRP	MRP1 MRP5	intestines, brain		[37,40,41,46,48]
		MRP2	intestines, liver, kidney, brain, placenta		
		MRP3	intestines, liver		
		MRP4	intestines, liver, kidney, brain		
	BSEP	BSEP	liver		[39]

## 3. Transporter-Mediated HDIs

### 3.1. HDIs That Improve Therapeutic Efficacy and/or Reduce Toxicity

Many herbs in combination therapies with chemical drugs exert synergistic and/or additive effects, and in some cases, alleviate side effects or toxicity of the concomitantly used drug. Many studies have shown that this may be related to the regulation of transporters by herbs and their derivatives, which can affect absorption, distribution, and excretion of substrates by inducing or inhibiting the activity of drug transporters, in order to achieve the effect of enhancing efficacy or reducing toxicity [31]. Here are some reported herbs that can increase drug efficacy or reduce drug toxicity mediated by transporters (Table 2).

#### 3.1.1. *Securidaca inappendiculata* Hassk.

*Securidaca inappendiculata* Hassk. is a traditional herbal medicine used to treat fractures, inflammation, and rheumatoid arthritis. Flavone derivatives are the main bioactive components promoting the anti-rheumatic properties of this plant [50,51].

Clinically, MTX is often used to treat rheumatoid arthritis, but in long-term treatment, it was found that because renal excretion constitutes the major elimination route of MTX, MTX treatment can cause nephrotoxicity in vivo, even at a very low dose [52]. Therefore, some researchers have wondered whether the combination of *Securidaca inappendiculata* Hassk. and MTX can be used to enhance efficacy and reduce the toxicity of MTX. Recent studies have found that a xanthone-rich fraction (XRF) in *Securidaca inappendiculata* Hassk. weakened MTX-induced proximal tubular edema. The experimental results show that XRF pre-treatment augmented MTX secretion into urine. XRF treatment significantly restored the suppressed OAT3 expression induced by MTX, as revealed by immunoblotting assay. OAT3 was extensively expressed in proximal renal tubules under normal conditions. MTX treatment diminished it greatly. The combined use of XRF totally reverts this trend. XRF promoted the excretion of MTX into urine by increasing the expression of OAT3. As a result, XRF attenuated MTX-induced edema of the proximal tubule. In vitro experiments showed that XRF restored the expression of OAT3 and enhanced the uptake capacity of MTX by HEK 293T cells, which indicated that XRF effectively protected MTX-induced renal secretion impairment by restoring the expression of OAT3. However, it is worth noting that the bioavailability of MTX was reduced to a large extent. Thus, it is worth paying attention to how to balance safety and efficacy in combined treatments [53].

#### 3.1.2. *Morinda officinalis* F.C. How.

*Morinda officinalis* F.C. How. *(Rubiaceae)* (MO), also known as “*Bajitian*” in Chinese, is one of the most famous herbs in China, South Korea, and Japan. In traditional Chinese medicine (TCM), MO is often used to treat various diseases, including male impotence [54], female infertility, fatigue, chronic rheumatism, and depression. Modern pharmacological experiments have shown that MO has a wide range of pharmacological activities, including antioxidant, analgesic, anti-inflammatory, anti-osteoporosis, antidepressant, and fertility-promoting activities [55,56]. MO has also been shown to help improve memory and treat Alzheimer’s disease [57,58,59]. Bajijiasu (BJJS) is one of the main bioactive compounds isolated from MO, which has a variety of pharmacological activities and therapeutic effects. Doxorubicin is a cancer chemotherapeutic drug and a substrate of P-gp. BJJS activated Nrf2, induced the expression of P-gp, enhanced the efflux activity of P-gp and reduced the toxicity of doxorubicin to cells [60]. Although the combination of MO and doxorubicin can reduce toxicity, it is still necessary to pay attention to detrimental HDIs due to the regulation of MO on P-gp [60].

#### 3.1.3. Other Herbal Active Ingredients

##### Flavonoids

Flavonoids have been proven to have anti-hypertension, diabetes, cardiovascular disease and other activities, and have been listed as the main components of drugs and health products, including troxerutin tablets and ginkgo biloba, which are also widely distributed in herbal foods, increasing the possibility of using them together with other drugs. It is necessary to consider the interaction between flavonoids and other drugs [61].

##### Rutin

Rutin is a flavonol that is abundant in plants such as passionflower, buckwheat, tea, and apple. It is an important nutritional component in food. Chemically, it is a glycoside, which is composed of the flavonoid glycoside quercetin and rutin disaccharide. It has many pharmacological activities, including antioxidant, cell protection, vascular protection, anticancer, neuroprotective, and cardioprotective activities [62]. Diclofenac is a widely used non-steroidal anti-inflammatory drug for the treatment of various inflammatory diseases. Although diclofenac is well absorbed orally, it can produce gastrointestinal diseases and cardiovascular complications. Studies have found that in rats, the therapeutic effect (anti-nociceptive and anti-inflammatory activity) of diclofenac is enhanced in the presence of rutin. Diclofenac is a substrate of BCRP, and rutin can inhibit the expression of BCRP. The research mechanism shows that rutin can enhance the intestinal permeability of diclofenac which may be related to the regulation of rutin on BCRP. Therefore, rutin has been found to be a promising drug candidate that can improve the efficacy of diclofenac through the expected HDIs. In the future, suitable diclofenac and rutin pharmaceutical compositions can be established with improved efficacy and better tolerance [63].

##### Quercetin

Quercetin is one of the most studied flavonoid compounds and has antioxidant and anti-aging effects. Most of these flavonoid compounds are in the form of glycosides, such as rutin, quercetin, hyperin, etc., and are widely present in berberine, hypericum, and other common plants [64]. Quercetin can inhibit the expression of P-gp [65,66].

Irinotecan is a semi-synthetic derivative of camptothecin, which has a growing clinical impact due to its effectiveness in colon, lung, pancreatic, cervical, and ovarian malignancies, with increased survival benefits for patients. However, it has the side effect of diarrhea which is associated with excessive bile excretion of its metabolite 7-ethyl-10-hydroxy camptothecin (SN-38). Pharmacokinetic studies showed that the absolute bioavailability of irinotecan in female Wistar rats pretreated with quercetin increased by 1.3 times compared with the control group. Mechanism studies showed that quercetin can increase the plasma concentration and reduce the bile levels of irinotecan and SN-38 by inhibiting intestinal P-gp activity, and improved the diarrhea caused by irinotecan [67]. These results suggest that the regulation of P-gp by quercetin in combination with anticancer drugs is likely to improve adverse reactions in patients, which is worthy of further clinical trials.

##### Apigenin

Apigenin is a flavonoid compound, which is widely distributed in natural plants and is present in vegetables and fruits, especially in celery. Compared with other flavonoids, apigenin has advantages due to its low inherent toxicity. Apigenin inhibits several drug transporters, such as OATP1B1, OATP1B3, and OAT1 [68]. Adefovir is an antiviral drug that can cause nephrotoxicity during treatment. Some studies have used apigenin and adefovir at the same time and found that apigenin effectively inhibited the activity of OAT1 in a dose-dependent manner. At the dose of 50 μM, apigenin significantly reduced the cytotoxicity of adefovir in MDCK-OAT1 cells and significantly increased cell viability from 50.6% to 112.62%. This showed that apigenin regulates OAT1 and can cause HDIs when used in combination with adefovir. When apigenin is used in combination with the substrate of OAT1, apigenin can be used as a renal protector [69].

##### Resveratrol

Resveratrol is a polyphenol found naturally in red grapes, mulberries, and peanuts. In addition to its well-known effects as a powerful antioxidant, resveratrol possesses extensive beneficial activities. These include anti-inflammatory, antiplatelet aggregation, anti-fibrotic, anti-allergic, and anti-aging actions. Resveratrol has been shown to exhibit profound multiorgan protective effects, including on the cardiovascular system, liver, and neurons, although many of the resveratrol-induced in vivo positive effects are only achieved at relatively high doses and long treatment periods, probably due to its extensive metabolism [70].

Resveratrol can up-regulate the expression of BCRP in the kidney. BCRP is mainly distributed in the apical membrane of tubules in the kidney and participates in the urinary clearance of organic anionic drugs. When resveratrol is combined with MTX, the ATPase activity of human BCRP increases, which activates the protective mechanism of the kidney, accelerates the clearance of MTX in urine and reduces nephrotoxicity [70]. Other studies have shown that resveratrol reduces the testicular toxicity of MTX by up-regulating the expression of MRP3 [71]. Different than the detoxification effect of resveratrol on MTX, some studies have investigated the mechanism of resveratrol in enhancing the toxicity of cisplatin. The experiments showed that resveratrol could reduce the absorption of glutamine by inhibiting the expression of the glutamine membrane transporter (ASCT2) and then enhancing the sensitivity of cells to cisplatin chemotherapy [72]. It can be seen that the regulation of transporters by resveratrol combined with drugs will protect the organs and promote the cytotoxicity of cancer cells, which is of significant benefit in clinical treatment.

##### Berberine

Berberine is an isoquinoline alkaloid present in many plants, including *Coptis chinensis*. It is an important component with pharmacological activity in Chinese herbal medicine. Berberine has a wide range of effects and can be used in the treatment of diabetes, inflammation, bacterial and viral infections, and cardiovascular diseases [73]. Metformin, a biguanide derivative, is extensively eliminated in the kidney as the parent drug (79–86% of an intravenous dose) via tubular secretion mediated by OCTs. As both metformin and berberine can be used for the treatment of diabetes, some studies have examined the pharmacokinetic interaction between metformin and berberine in vivo after oral administration. Uptake transporters such as OCT1 located in the liver and OCT2 located in the kidney transport berberine from the blood into liver or kidney tubular epithelial cells and play an important role in the tissue distribution and elimination of berberine. The study found that metformin increased the C_max_ and AUC_0–4h_ of berberine by 33.1% and 57.7%. The cell inhibition test revealed that metformin (≥ 1 and ≥ 0.3 mM) decreased the berberine concentration in MDCK-rOCT1 and MDCK-rOCT2 cells. So the co-administration of metformin probably inhibited the uptake of berberine by the liver and kidney and then increased the plasma concentration. Therefore, metformin combined with berberine might be beneficial in the treatment of diabetes [74,75]. 

### 3.2. HDIs That Produce Adverse Reactions

Although we can enhance the efficacy of chemical drugs or reduce their toxicity by regulating the expression of transporters using herbs (Table 2), we cannot ignore the possible additional adverse reactions caused by HDIs due to the regulation of herbs on transporters which will have a negative impact on clinical treatment. Several cases of HDIs due to transporters and the corresponding mechanisms were identified in the literature in order to confirm the mechanism and possible risk of HDIs caused by transporters and are shown in Table 3.

**Table 2:** Effects of herbal extracts or components on transporters and the beneficial impact of HDIs.

Herbs	Chemical Composition/Drugs		Methods	Transporter Effect	Affected Drugs/Herbs	Reactions	References
*Securidaca inappendiculate* Hassk.	xanthone-rich fraction		in vivo, rats; in vitro, HEK 293T cells	OAT3 ↑	methotrexate	Promote methotrexate excretion and reduce proximal tubular edema	[53]
*Morinda officinalis* F.C. How.	bajijiasu		in vivo, mice; in vitro, HepG2 cells	P-gp ↑	doxorubicin	Promote doxorubicin efflux and reduce cytotoxicity	[60]
Herbal active ingredients	flavonoids	rutin	in vivo, rats;	BCRP ↓	diclofenac	Increase the intestinal permeability of diclofenac and improve the curative effect	[62,63]
		quercetin	in vivo, rats; in vitro, Caco-2 cells (HTB-37)	P-gp ↓	irinotecan	Increase the blood concentration of irinotecan, reduce the level of irinotecan in bile and improve the diarrhea caused by irinotecan	[67]
		apigenin	in vivo, rats; in vitro, MDCK-OAT1 cells	OAT1↓	adefovir	Reduced cytotoxicity	[69]
	resveratrol		in vivo, rats	BCRP ↑	methotrexate	Promotes the clearance of methotrexate in urine and reduces nephrotoxicity	[70]
			in vivo, rats; in vitro, PC3 cells	MRP3↑		Promote MTX efflux	
			in vitro, C3A, SMCC7721 and LO2 cells	ASCT2 ↓	cisplatin	Glutamine metabolism is inhibited, which promotes the sensitivity of human liver cancer cells to cisplatin chemotherapy	[72]
	metformin		in vivo, rats; in vitro, HEK293-OCT1 and -OCT2 cells	OCT1 ↓	berberine	Increase the plasma concentration of berberine.	[75]
				MATE1 ↓			

↑, inducing; ↓, inhibitory.

#### 3.2.1. Red Ginseng

The active components of ginseng mainly include ginsenosides, polysaccharides, alkaloids, peptides, and phenolic compounds, which have anti-inflammatory, antidiabetes, anticancer, anticardiovascular, and cerebrovascular disease activity, and plays an important role in traditional Chinese herbal medicine [76]. Red ginseng is the root and rhizome of raw ginseng after steaming and drying under high temperatures and high pressure. Although it is different from the processing technology for ginseng, it is also defined as “ginseng” and has more abundant pharmacological activities [77].

Red ginseng contains a variety of bioactive components, including flavonoids, phenolic acids, and ginsenosides. It is reported that red ginseng extract (RGE) has an anti-diabetes role by preventing mitochondrial damage and inhibiting intracellular inflammation [78]. As red ginseng can reduce oxidative stress damage induced by hyperglycemia, some diabetic patients take red ginseng and its preparations as adjuvant treatment when taking prescription drugs for diabetes, which may produce adverse HDIs. It is reported that repeated RGE administration for one week can increase the expression of OCT1 mRNA in rat intestine and promote the absorption of metformin [79]. This is related to the expression site of uptake transporter OCT1 in the intestine. OCT1 is located in the apical membrane of intestinal cells. RGE increases the intestinal uptake and transport of metformin by increasing the level of OCT1 protein, thus increasing the plasma concentration of metformin. Metformin can cause diarrhea, and an increase in metformin blood concentration may cause more serious side effects; therefore, cautious is necessary when combined with red ginseng.

Experiments have been carried out to study the effect of RGE on efflux transporters. One week after intragastric administration of RGE (1.5 g/kg/d), the mRNA and protein levels of MRP2, BSEP, and P-gp in rat liver were monitored. It was found that repeated RGE administration reduced the expression of MRP2. The pharmacokinetics of MTX, the substrate of MRP2 probe, was further studied in rats. It was shown that bile excretion of MTX was reduced and the area under the plasma drug time curve was increased by 1.5 times in the RGE repeated administration group compared with the control group [80]. In the liver, MRP2 is located in the apical membrane of hepatocytes and is responsible for pumping drugs in the liver into bile for clearance. Long-term administration of red ginseng can reduce the expression of MRP2 and the clearance of MTX, resulting in increased exposure to drugs in the body. MTX, an immunosuppressant and antitumor drug commonly used in the clinic, has a narrow treatment window. The change in blood drug concentration caused by the use of herbal medicine requires further attention [81]. Long-term multi-dose administration of RGE can reduce the hepatobiliary excretion of MRP2-specific substrates, thus inducing unnecessary HDIs, which requires therapeutic drug monitoring and determination of the safety of clinical combinations.

#### 3.2.2. *Radix astragali*

*Radix astragali* is a common Chinese herbal medicine. Its effective components include saponins, isoflavones, polysaccharides, and various trace elements. It has the functions of protecting the liver, diuresis, analgesia, sedation, and enhancing body immunity [82]. As *Radix astragali* can activate human immune function, it is mostly used as a dietary supplement in daily life or as a clinical adjuvant.

Experiments have shown that *Radix astragali* and its three main active components, including astragaloside IV, calyx anthocyanin, and formononetin, can induce the expression of P-gp and BCRP and increase the efflux function mediated by P-gp and BCRP by activating the Nrf2 mediated signal pathway [83]. A study on the effect of astragaloside IV on the pharmacokinetics of omeprazole in vivo was carried out in male rats. It was found that following pretreatment with astragaloside IV (100 mg/kg/d for 7 days), the C_max_ and AUC_0–t_ of omeprazole (2 mg/kg) in rats were 74% and 61% of those in the control group, respectively. A transport study in the Caco-2 cell line found that after pretreatment with astragaloside IV, the efflux ratio of omeprazole in the trans Caco-2 cell transport model increased from 1.73 to 2.67. This suggests that astragaloside IV may reduce the absorption of omeprazole in the intestine by inducing the efflux activity of P-gp, in order to reduce the bioavailability of omeprazole in vivo [84]. In addition, experiments have proven that when puerarin is used in combination with astragaloside IV, it also reduces the exposure in vivo due to the induction of P-gp by astragaloside IV [85]. These results suggest that potential HDIs may occur when *Radix astragali* or its active components are used in combination with other drugs, which are P-gp and BCRP substrates. Whether these potential HDIs will reduce the efficacy or increase the induced toxicity needs to be further verified in the clinic and by research.

#### 3.2.3. St John’s Wort

St. John’s wort (SJW), also known as *Hypericum perforatum* L., is a perennial herb of Hypericum. and has the effect of sedation and hypnosis. Its antidepressant effect has been confirmed by many studies. It is the preferred herbal medicine for the treatment of depression and anxiety in European countries and America [86].

Studies have shown that SJW can induce P-gp activity and produce clinical HDIs when used together with P-gp substrates [87]. SJW increased P-gp expression and enhanced drug excretion in peripheral blood lymphocytes from healthy volunteers [88]. The pharmacokinetic effects of SJW on digoxin were evaluated by chemiluminescence immunoassay. Eighteen young volunteers (9 women and 9 men) received SJW extract (300 mg, 3 times/d) and other control drugs for 14 days, followed by digoxin (0.25 mg), and blood samples were tested continuously within 24 h. The results showed that compared with the control group, SJW administration led to a significant decrease in the C_max_ and AUC of digoxin, which could lead to more clinically significant P-gp-mediated HDIs [88]. In another single-blind, placebo-controlled parallel study, 25 healthy volunteers (12 women, 13 men) received a continuous load of oral digoxin (0.25 mg, twice a day) for two days, followed by 0.25 mg of digoxin daily up to day 15. Volunteers took SJW extract and placebo from day 6 to day 15, and blood samples were collected on day 5 (before combined administration), day 6, and day 15 for pharmacokinetic analysis. The experiment showed that the AUC and C_max_ of digoxin in the SJW extract group decreased by 33% and 26%, respectively, compared with the placebo group after day 15 of oral administration, and this absorption inhibition effect was continuously enhanced compared with day 6 [89,90]. These findings showed that the pharmacokinetic effect of SJW extract on digoxin is time-dependent, and this phenomenon is related to the induction of active expression of P-gp.

Cyclosporine (CSP), an important immunosuppressant with a narrow therapeutic window, is widely prescribed to prevent allograft rejection in transplant patients and to treat rheumatoid arthritis and psoriasis. The use of CsA in transplantation medicine has been shown to cause a number of toxic cellular side effects, including nephrotoxicity, hepatotoxicity, neurotoxicity, and myocardial toxicity [91]. In some clinical cases, it was also found that taking SJW during CSP treatment in organ transplant recipients induced a reduction in the concentration of CSP in plasma to the sub-therapeutic level, resulting in acute rejection [4,5,6]. SJW can not only promote the expression of P-gp, but also induce the activity of CYP3A4 [7]. CSP is the common substrate of CYP3A4 and P-gp. SJW-mediated HDI may be the result of the interaction between transporters and metabolic enzymes. SJW reduces the absorption of CSP in the intestine by increasing the expression of intestinal P-gp, which may indirectly prolong the enzymatic reaction of CYP3A4 to CSP and enhance the first pass effect of the drug. Due to the large mucosal surface area and rich blood flow of the small intestine, its unique structural characteristics determine that the small intestine is the main place for food digestion and absorption, while the expression of P-gp is abundant in the small intestine, and the retention time of oral drugs in the intestine is longer, which increases the opportunity for this interaction.

#### 3.2.4. *Polygonum cuspidatum*

*Polygonum cuspidatum* (PC), a medicinal plant of the Polygonaceae family of eudicots, has the effects of clearing away heat and detoxification, promoting dampness, and resolving phlegm. It is an important component of traditional Chinese herbal medicine. Modern pharmacological research has shown that PC has anti-inflammatory and antioxidant effects and can be used to treat cancer and other inflammatory diseases [92,93].

Carbamazepine (CBZ) is the substrate of P-gp and MRP2. These two efflux transporters are distributed in the basolateral membrane of brain endothelial cells and play a protective role in the BBB. They are considered to be one of the main reasons for CBZ resistance and recurrent seizures. It has been reported that PC can significantly increase the systemic exposure and brain drug concentration of CBZ by inhibiting the activities of MRP2 and CYP3A [94]. In patients treated with CBZ, attention should be paid to avoiding toxicity when combined with PC. On the contrary, for patients with resistance to CBZ, combined treatment with PC may help to reverse resistance. Careful monitoring of the combination of PC and key drugs (substrates of CYP3A4 and/or MRP 2) is very important in the clinic.

#### 3.2.5. *Rheum palmatum*

*Rheum palmatum* (RP) is a commonly used herb in clinical Chinese medicine which has the functions of antibacterial and anti-inflammatory, cholagogic and liver protection, heat-clearing, and detoxification. The major constituents of rhubarb are a variety of phenolic compounds, such as anthraquinone derivatives, dianthrones, stilbenes, polyphenols, flavonoids, and chromones. Recent pharmacokinetic studies of RP have revealed that the anthraquinones were predominantly present as glucuronides and sulfates in the blood, and they are also putative substrates of MRPs and OATs [95,96].

Phenytoin (PHT), a widely used antiepileptic with a narrow therapeutic window, follows nonlinear pharmacokinetics, and thus therapeutic drug monitoring is usually recommended during its use. The adverse reactions of PHT include drowsiness, dysarthria, tremor, and cognitive difficulties. PHT has been reported to be a substrate of P-gp and MRP2, whose expression determines the PHT level in the brain. Some studies have observed the acute and chronic effects of RP on the pharmacokinetics of phenytoin sodium in rats. The results showed that when combined with RP, it could significantly reduce the C_max_ and AUC of phenytoin sodium in rats. Cell experiments showed that RP could significantly induce the activity of P-gp and mediate the efflux of phenytoin sodium, but inhibit the activity of MRP2. In an experiment, it was found that the oral bioavailability of phenytoin sodium decreased after combined administration in rats. It can be inferred that this was achieved by enhanced expression of P-gp activity by *RP* [97]. Studies have found that RP can activate the functions of P-GP and CYP 3A, and significantly reduce the C_max_ and AUC_0-t_ of CSP in rats. RP–CSP interaction might pose a nonnegligible hidden risk of allograft rejection for transplant patients. RP might lead to potential herb-drug interactions with substrate drugs of P-gp and/or CYP 3A4, which might result in therapeutic failure [91].

#### 3.2.6. *Dioscorea bulbifera* L.

*Dioscorea bulbifera* L. (DB) is a clinical Chinese herbal medicine with a wide range of pharmacological activities. The extract from DB rhizomes contains a variety of components, including diterpene lactones, steroidal saponins, flavonoids, alkaloids, and micronutrients. It has an antitumor effect and can inhibit cervical cancer and liver cancer [8].

Pirarubicin (THP) is an anthracycline drug that interferes with DNA synthesis in tumor cells. It is widely used in the treatment of several tumors. However, the clinical application of THP is severely limited due to its severe cardiotoxicity, which is irreversible, and the drug can accumulate. Some studies have evaluated the effects of the combination of DB and THP on liver and heart injury in mice, the accumulation of THP, and the localization and expression of P-gp and MRP2 to study the mechanism of HDIs between DB and THP. The results showed that when DB extract was combined with THP, the expression of P-gp and MRP2 was down-regulated, the excretion of THP was reduced, and then accumulated in vivo, aggravating cardiotoxicity. Therefore, it is necessary to monitor cardiotoxicity in the clinic when DB extract and THP are administered together [98].

#### 3.2.7. Other Herbal Active Ingredients

##### Flavonoids

Paracetamol (acetaminophen) is a para-aminophenol derivative and possesses analgesic and antipyretic activities similar to aspirin. Studies have evaluated the effect of the flavonoid quercetin on the pharmacokinetics of paracetamol in rats. Paracetamol (100 mg/kg) combined with quercetin (5, 10, 20 mg/kg) was administered by gavage once a day for 21 days, and blood samples were taken for analysis. The results showed that quercetin could increase the plasma exposure of paracetamol in a dose-dependent manner. After combined administration of quercetin (20 mg/kg), the AUC of paracetamol increased from 26.44 ± 6.79 to 64.47 ± 6.80 mg h/mL, the half-life in vivo was prolonged, and the clearance rate was reduced. In the non-everted rat gut sac method, the absorption of paracetamol was increased in the presence of quercetin. As mentioned above, quercetin can inhibit the expression of P-gp. Paracetamol is metabolized by glucuronidation (40–67%) and sulfation (20–46%) into inactive and harmless metabolites. Quercetin has been reported to be a potent inhibitor of the production of both sulfate and glucuronide conjugates of paracetamol in cultured cells. These findings suggest that quercetin might inhibit P-gp and the metabolism of paracetamol, thereby increasing systemic exposure to paracetamol [99]. This suggests that quercetin can inhibit P-gp-mediated paracetamol transport and increase systemic exposure to drugs. The excessive accumulation of drugs in the body may induce and aggravate the hepatotoxicity of paracetamol. Thus, careful dosing is needed when paracetamol is co-administered with quercetin.

##### Sinapic acid

Sinapic acid (SA) is a phenolic acid, which is abundant in human foods, such as berries, citrus fruits, nuts, coffee and tea, and whole grains, as well as traditional herbs rich in erucic acid. It is used as a sedative, anticonvulsant, antidepressant, and antiepileptic drug (mustard, cabbage, black mustard, pyrethrum, kidney-shaped sweet potato, mistletoe, corn) [60,100,101,102].

The antiepileptic drug CBZ is effective in the treatment of seizures, convulsions, trigeminal neuralgia, and manic depression, but long-term use of CBZ can lead to liver injury. In patients with epilepsy who need long-term treatment, clinicians should provide liver protective dietary supplements. However, due to the narrow treatment window of CBZ, this may lead to potential HDIs. Studies have shown that CBZ is an effective inducer of various drug metabolic enzymes, such as CYP450 3a and 2b in the liver, and the metabolism of CBZ is related to transporters P-gp and MRP-2 [103]. A study using male Wistar rats was carried out to investigate the pharmacokinetic effect of SA on CBZ. It was found that the blood concentration of CBZ after SA pretreatment was higher than that without pretreatment. SA significantly inhibited the metabolism of CBZ in the liver mediated by CYP3A2 and CYP2C11, significantly inhibited the efflux of CBZ by intestinal P-gp, increased intestinal absorption, and improved the absorption rate of CBZ. This greatly increased the probability of liver injury. However, further studies are needed to determine the clinical relevance of these observations. Therefore, when taking CBZ, foods containing SA or traditional herbs should be used with caution [104].

**Table 3:** Effects of herbal extracts or components on transporters and HDIs that produce adverse reactions.

Herbs	Chemical Composition	Methods	Transporter Effect	Affected Drugs	Reactions	References
Red ginseng	red ginseng extract (RGE)	in vivo, rats; in vitro, HEK293 cells	OCT1 ↑	metformin	Increase the blood concentration of metformin, easy to cause diarrhea	[79]
		in vivo, rats;	MRP2 ↓	methotrexate	Bile excretion is reduced, methotrexate clearance is reduced, and burst leakage in the body is increased, prone to side effects	[80]
*Radix astragali*	astragaloside IV	in vivo, rats; in vitro, Caco-2 cell line	P-gp ↑	omeprazole	Decrease the absorption of omeprazole	[84]
St John’s wort	-	in vivo, human	P-gp ↑	digoxin	Reduce the absorption of digoxin in the intestine and reduce the bioavailability of drugs	[89,90]
		in vivo, human		ciclosporin	Reduce the absorption of ciclosporin in the intestine and enhance the first pass effect of drugs, produce acute rejection	[4,5,6]
*Polygonum cuspidatum*	-	in vivo, rats in vitro, LS 180 and MDCKII–MRP 2 cell lines	MRP2↓	carbamazepine	Increased systemic exposure to carbamazepine, causing carbamazepine resistance and recurrent seizures	[94]
*Rheum palmatum*	-	in vivo, rats; in vitro, LS 180 and MRP-2-overexpressing MDCK II cell lines	P-gp ↑	phenytoin	Promote the efflux of phenytoin sodium, reduce the bioavailability and reduce the curative effect	[97]
			MRP2 ↓	drugs with MRP2 as substrate	Increase the amount of drug leakage in the body and increase the toxicity	[97]
		in vivo, rats	P-gp ↑	cyclosporine	Reduce the systemic exposure of CSP and increase the risk of allogeneic rejection	[91]
*Dioscorea bulbifera* L.	extract from *Dioscorea bulbifera* L. rhizomes	in vivo, mice	P-gp ↓ MRP2 ↓	pirarubicin	Reduce pirarubicin exclusion, pirarubicin accumulation in the body and aggravate cardiotoxicity	[98]
herbal active ingredients	quercetin	in vivo, rats; in vitro, Caco-2 cells	P-gp ↓	paracetamol	Increase blood drug concentration of paracetamol and aggravate liver toxicity	[99]
	Sinapic acid	in vivo, rats	P-gp ↓	carbamazepine	Inhibit the excretion of drugs in the intestine and metabolism in the liver, increase the absorption of carbamazepine and enhance liver injury	[97,103,104]

↑, inducing; ↓, inhibitory.

## 4. Discussion

Over the past decades, the use of herbal medicine as an important part of multi-component therapy has steadily increased. Approximately 80% of people in Asian countries use herbal medicine to promote health and treat common diseases, such as inflammation, pain, heart disease, liver cirrhosis, and central nervous system diseases [105]. Many people use herbal medicine as a dietary supplement for long periods. With the increased use of herbal medicine, the probability of HDIs is greatly increased. A variety of complex drug treatment methods increase the possibility of HDIs. It is necessary to understand the occurrence mechanism of HDIs for rational clinical medication. 

Many HDIs are mediated by transporters and are also closely related to changes in the activity and expression of drug metabolic enzymes [106]. Studies have shown that the expression of P-gp and cytochrome P450 (CYP450) enzymes are jointly regulated by the pregnane X receptor (PXR) and constitutive androstane receptor (CAR) [106,107]. Most drugs with a high affinity for transporter P-gp are also substrates of the CYP3A enzyme system. There are also reports that BCRP can be coupled with metabolic enzymes to accelerate the removal of foreign bodies in the body and promote the excretion of metabolites catalyzed by phase I and II metabolic enzymes into bile [3]. Drug transporters and metabolic enzymes may interact synergistically with the dynamic process of drugs in the body. The potential interaction between metabolic enzymes and efflux transporters on drug elimination may affect drugs in some cases. For example, rhubarb can activate P-gp and CYP3A4 to reduce the bioavailability of CSP [108,109,110]. St. John’s wort regulates nuclear receptors (mainly PXR) at the transcriptional level, induces the expression of P-gp and CYP3A4, reduces the absorption of CSP in the intestine and increases the metabolism of enzymes, directly or indirectly leading to enhancement of the first pass effect of the drug [26]. We need to study both transporters and CYP450 at the same time and understand which is the main factor causing the results in order to make the mechanism more accurate and complete when considering the results of HDIs.

Although there are a large number of reports on HDIs, due to the complex nature of TCM and poor quality control, the test system has different responses to TCM exposure and different research designs. Many results are contradictory, which may cause confusion, and it is usually impossible to predict the degree or clinical significance of HDIs. For example, there are studies that have found no interaction between *ginkgo folium* extract and CYP2C9 probe substrate observed in vivo, contrary to the CYP2C9 inhibition observed in vitro [111]. In addition, *Ginkgo folium* extract induces CYP2B in vivo, but inhibits CYP2B in vitro. Orally administered ginkgo folium extract only affects the pharmacokinetics of oral but not intravenously administered nifedipine in rats [112]. Metabolism of herbal medicines in the body may cause differences between in vivo and in vitro effects. The absorption and distribution of herbal components may lead to different in vivo effects [110]. In order to avoid these problems, we require further improvements and efforts in many different aspects. For example, the market for plant supplements should strictly implement standardized production practices and reliable label information to ensure the quality of herbal medicines. The test system should be selected according to the advantages and disadvantages of different systems, and the selected system should be able to reliably predict clinical HDIs according to the obtained data. For example, human tissue and cell line systems are used for in vitro research, while animal species most similar to humans, especially humanized animals, are more suitable for in vivo HDIs preclinical research. If there are specific authoritative guidelines to provide recommendations for HDIs research, these would be of great help in solving these problems to optimize the research design according to clinical relevance.

It is known that HDIs can be beneficial or harmful. We can intentionally use predictable HDIs to increase therapeutic efficacy or reduce side effects. Furthermore, we should try to avoid harmful HDIs. Quantitative assessments of the HDI potential and more clinically relevant research on an investigational drug should be performed so that the results can be used to predict whether dosage adjustments, prescribing modifications, or other measures are needed to reduce risk and avoid undesired consequences. We should focus on educating natural health product retailers and pharmacists on potential HDIs, and improve clinical risk management methods. We should also consider the treatment window of herbal medicine. Many herbs’ treatment window is very narrow, such as *Coptidis rhizome*. Serious HDIs may also lead to controversy regarding the safety of herbal medicine [110]. During the development of drugs and herbal medicines, risk-benefit evaluation should be carried out to improve the safety of clinical medication by formulating standard guidelines for combined medication. Although the safety of herbal medicines is becoming more and more valued, only the Chinese regulatory authority has explicitly considered herb-drug interactions in their DDI guidance, while the FDA, EMA, and JPDMA do not mention herbal medicines. Efforts of various regulatory authorities are needed: such as formulating standard guidelines for the combined use of herbal medicines and drugs, carrying out risk-benefit evaluation, and improving clinical drug safety.

## Data Availability

Data sharing not applicable.

## Abbreviations

ABC	ATP-binding cassette
ASCT2	glutamine membrane transporter
BBB	blood-brain barrier
BCRP	breast cancer resistance protein
BCSFB	blood-cerebrospinal fluid barrier
BECs	brain endothelial cells
BJJS	bajijiasu
BSEP	the bile salt export pump
CAR	constitutive androstane receptor
CBZ	carbamazepine
CIA	collagen-induced arthritis
CP	choroid plexus
CSF	cerebrospinal fluid
HDIs	herb–drug interactions
MATEs	mammal multidrug and toxin extrusion proteins
MO	*Morinda officinalis* F.C. How.
MRPs	multidrug resistance-associated proteins
MTX	methotrexate
OATs	organic anion transporters
OATPs	the organic anion transporting polypeptides
OCTs	organic cation transporters
OCTNs	carnitine transporters
PC	*Polygonum cuspidatum*
PEPTs	peptide transporters
PHT	phenytoin
P-gp	P-glycoprotein
PXR	pregnane X receptor
RGE	red ginseng extract
RP	rheum palmatum
SA	sinapic acid
SLC	solute carrier
SJW	St. John’s wort
THP	pirarubicin
XRF	xanthone rich fraction
